# Different effection of p.1125Val>Ala and rs11954856 in APC on Wnt signaling pathway

**DOI:** 10.18632/oncotarget.20106

**Published:** 2017-08-05

**Authors:** Fei-Feng Li, Zhi-Xun Zhao, Peng Yan, Song Wang, Zheng Liu, Qiong Zhang, Xiao-Ning Zhang, Chang-Hao Sun, Xi-Shan Wang, Gui-Yu Wang, Shu-Lin Liu

**Affiliations:** ^1^ Systemomics Center, College of Pharmacy, and Genomics Research Center (State-Province Key Laboratories of Biomedicine-Pharmaceutics of China), Harbin Medical University, Harbin, China; ^2^ Translational Medicine Research and Cooperation Center of Northern China, Heilongjiang Academy of Medical Sciences, Heilongjiang, China; ^3^ Department of Colorectal Surgery of the Second Affiliated Hospital, Harbin Medical University, Harbin, China; ^4^ Department of Colorectal Surgery, Cancer Institute and Hospital, Chinese Academy of Medical Sciences and Peking Union Medical College, Beijing, China; ^5^ Department of Antibiotics, Heilongjiang Province Food and Drug Inspection Testing Institute, Harbin, China; ^6^ Department of Nutrition and Food Hygiene, Public Health College, Harbin Medical University, Harbin, China; ^7^ Department of Microbiology, Immunology and Infectious Diseases, University of Calgary, Calgary, Canada

**Keywords:** colorectal cancer, APC, mutation, Wnt/β-catenin signaling pathway, gene expression

## Abstract

Colorectal cancer (CRC) is among the most common and fatal forms of solid tumors worldwide and more than two thirds of CRC and adenomas patients have *APC* gene mutations. APC is a key regulator in the Wnt/β-catenin signaling pathway but its roles in CRC remains to be elucidated. In this study, we compared *APC* genes between CRC patients and controls to determine possible associations of nucleotide changes in the *APC* gene with the pathways involved in CRC pathogenesis. All participants received physical and enteroscopic examinations. The *APC* gene was sequenced for 300 Chinese Han CRC patients and 411 normal controls, and the expression levels of genes in the signaling pathway were analyzed using Western Blotting. Statistical analyses were conducted using SPSS (version 19.0) software. We found that rs11954856 in the *APC* gene was associated with colorectal cancer and could increase the expression levels of *APC*, *β-catenin*, *TCF7L1*, *TCF7L2* and *LEF1* genes in the pathway in the CRC patients, demonstrating the involvement of APC in the pathological processes leading to CRC.

## INTRODUCTION

Colorectal cancer (CRC) is one of the most common and fatal forms of solid tumors in both men and women [1, 2]. Most CRC cases are sporadic, with about 15–25% having a family history [3, 4] and 5% diagnosed with inherited CRC syndrome [5]. Many genetic factors have been reported for their associations with the CRC syndrome [6]. For example, instability of the chromosomes and changes in the CpG island methylator phenotype may cause defects in the pathways, leading to CRC [6–8]. To date, a large number of somatic mutations have been identified in numerous genes for their involvement in colorectal cancer, such as *KRAS* [9, 10], *PIK3CA* [11], *BRAF* [12], *MYH11* [13], and *PARK2* [14]. Additionally, multiple mutations concurrently appearing in a gene may result in marked predisposition to CRC, such as those in the adenomatous polyposis coli (*APC*) gene, which is associated with familial adenomatous polyposis disease (FAP) [15]. Other examples of connections between CRC and genomic variations include mutations in genes such as *MSH2* [16], *MLH1* [17], *PMS1* [18], *TGFBR2* [19] etc, which are associated with hereditary nonpolyposis colorectal cancer (HNPCC), and those in the *MUTYH* gene, which are associated with autosomal recessive adenomatous polyposis [20]. However, the exact functions of the variations or mutations in these genes remain largely unknown.

*APC* is a key regulator in the Wnt/β-catenin signaling pathway, modulating the quantity ofβ-catenin in the cells. It plays key roles in several fundamental life processes, such as cell division, signal transduction, and tumor suppression [21, 22]. Several mutations and deletions in the APC gene and methylation in its promoter [23] have been described in FAP, and more than two thirds of CRC and adenomas patients have APC gene mutations [23–25]. Additionally, APC gene mutations have also been reported in tumors of other tissues and organs, such as the liver [4], stomach [5, 8, 26], lung [27], breast [28], and brain [29], further calling for the elucidation of the roles of these genetic changes in carcinogenesis [21, 30, 31].

In a previous study, we identified a novel mutation (p.1125Val>Ala) in the APC gene, which is associated with FAP and sporadic cases of CRC [32]. The polyposis syndrome is one of the most common syndromes associated with CRC [29, 30]. However, as many CRC cases do not present polyposis, and the associations between gene changes and the disease are still not established even though recent technical advances have demonstrated an apparent inheritance pattern of CRC [31, 33], contributions of specific changes in the *APC* gene to CRC have not been fully documented.

In this work, we profiled nucleotide changes of the *APC* gene and found that the SNP rs11954856 was associated with CRC. This SNP increased expression levels of genes in the Wnt/β-catenin signaling pathway in the CRC cancer patients, including *APC*, *β-catenin*, and transcription factor genes *TCF7L1*, *TCF7L2*, *LEF1* in the pathway. Of particular significance, the p.1125Val>Ala mutation of the *APC* gene in the CRC cancer patients increased expression levels of not only genes downstream of *APC* in the Wnt/β-catenin signaling pathway but also a gene upstream of *APC*, *GSK-3β*, for feedback, providing further evidence indicating the involvement of the *APC* gene in the pathogenesis of CRC.

## RESULTS

### Clinical data

The clinical diagnosis was confirmed by three specialists in CRC in the Second Affiliated Hospital of Harbin Medical University, Harbin, China. There was no history of other systemic abnormalities of these CRC patients or previous tumor or familial history of tumor. All the CRC patients (n = 300, male 183, female 117, the min and max age were 16 and 87 respectively, and the average age was 58.59 years) and unrelated controls (n = 411, male 256, female 155, the min and max age were 50 and 70 respectively, and the average age was 59.39 years) were recruited specifically for this study, and there were no statistical differences in gender or age composition between the two groups (Supplementary Table 1).

### SNP gene analyses

Using standard protocols, we extracted the genomic DNA from the peripheral blood leukocytes [34] and sequenced the APC gene to detect SNPs and test the hypothesis that germline common genetic variants in the gene may be associated with the susceptibility to CRC. We analyzed the SNPs distributed on the APC gene from the NCBI database (http://www.ncbi.nlm.nih.gov/) and eventually chose six of them to focus on, including rs11241185, rs11954856, rs2019720, rs2229992, rs2431238 and rs2289484 (Supplementary Figure 1A), based on the finding that the genetic heterozygosity of these six SNPs was high (Supplementary Figure 1B).

### Polymorphism-disease association analyses

To test the hypothesized associations between *APC* variations and CRC, we conducted SNP analyses and found that the variant rs11954856 in the APC gene was associated with the risk of CRC in the Chinese Han population (Tables 1 and 2). We further analyzed the genotype frequencies in the CRC and control groups by three genetic models (allelic, dominant and recessive) and found that the variant rs11954856 was associated with the risk of CRC in allelic and dominant models (Table 3). On the other hand, we did not find statistical significance in the other analyzed APC gene SNPs rs11241185, rs2019720, rs2229992, rs2431238 and rs2289484 between the CRC and control groups (data not shown). We conducted the Hardy-Weinberg equilibrium test for the CRC and controls and the result was in line with equilibrium (Table 4). We also compared the genotype frequency of the rs11954856 in the CRC, control groups and the data from the HapMap HCB population, and the frequency in the control group was more consistent with the data from the HapMap HCB population (Table 5).

**Table 1:** The genotype and allele frequency of rs11954856 variations in 300 Chinese Han sporadic colorectal cancer patients and 411 non-CRC controls

*Group*		*Genotype frequency (%)*			*Allele frequency (%)*	
Genotype		G/G	G/T	T/T	G	T
CRC	300	179(59.7)	105(35.0)	16(5.3)	463(77.2)	137(22.8)
Controls	411	283(68.9)	116(28.2)	12(2.9)	682(83.0)	140(17.0)

**Table 2:** rs11954856 variation within *APC gene* associated with risk of sporadic colorectal cancer in Chinese populations

*Variations*	*Type*	*Pearson Chi-square*				*Risk*		
		Value	Min count^a^	df	Asymp. Sig.	OR	*95%CI-Up*	*95%CI-Low*
rs11954856	Genotype	7.381^a^	11.81	2	**0.025**	--	--	--
	Allele	7.443^a^	116.88	1	**0.006**	0.694	0.533	0.903

a: The minimum expected count; b: Not assuming the null hypothesis; c: Using the asymptotic standard error assuming the null hypothesis; d: Based on normal approximation.

**Table 3:** SNP rs11954856 variation within *APC gene* associated with risk of sporadic colorectal cancer in allelic and dominant model

*Value*	*Allelic model*	*Dominant model*	*Recessive model*
***ChisQ***	7.443	6.436	2.671
***P***	0.0064	0.0112	0.1022

**Table 4:** The CRC and controls groups were in line with Hardy-Weinberg equilibrium

*Group*		*Genotype frequency (%)*			*H-W equilibrium testing*		
Genotype		G/G	G/T	T/T	0 (HET)	E (HET)	P
CRC	300	179(59.7)	105(35.0)	16(5.3)	0.3500	0.3524	0.8708
Controls	411	283(68.9)	116(28.2)	12(2.9)	0.2822	0.2826	1.0000

**Table 5:** The frequency in control group was more consistent with the data from the HapMap HCB population

*Group*	*Genotype frequency (%)*		
Genotype	G/G	G/T	T/T
CRC	0.597	0.350	0.160
Controls	0.689	0.282	0.029
HCB data	0.682	0.295	0.023

### Gene expression analysis

**Figure 1:**
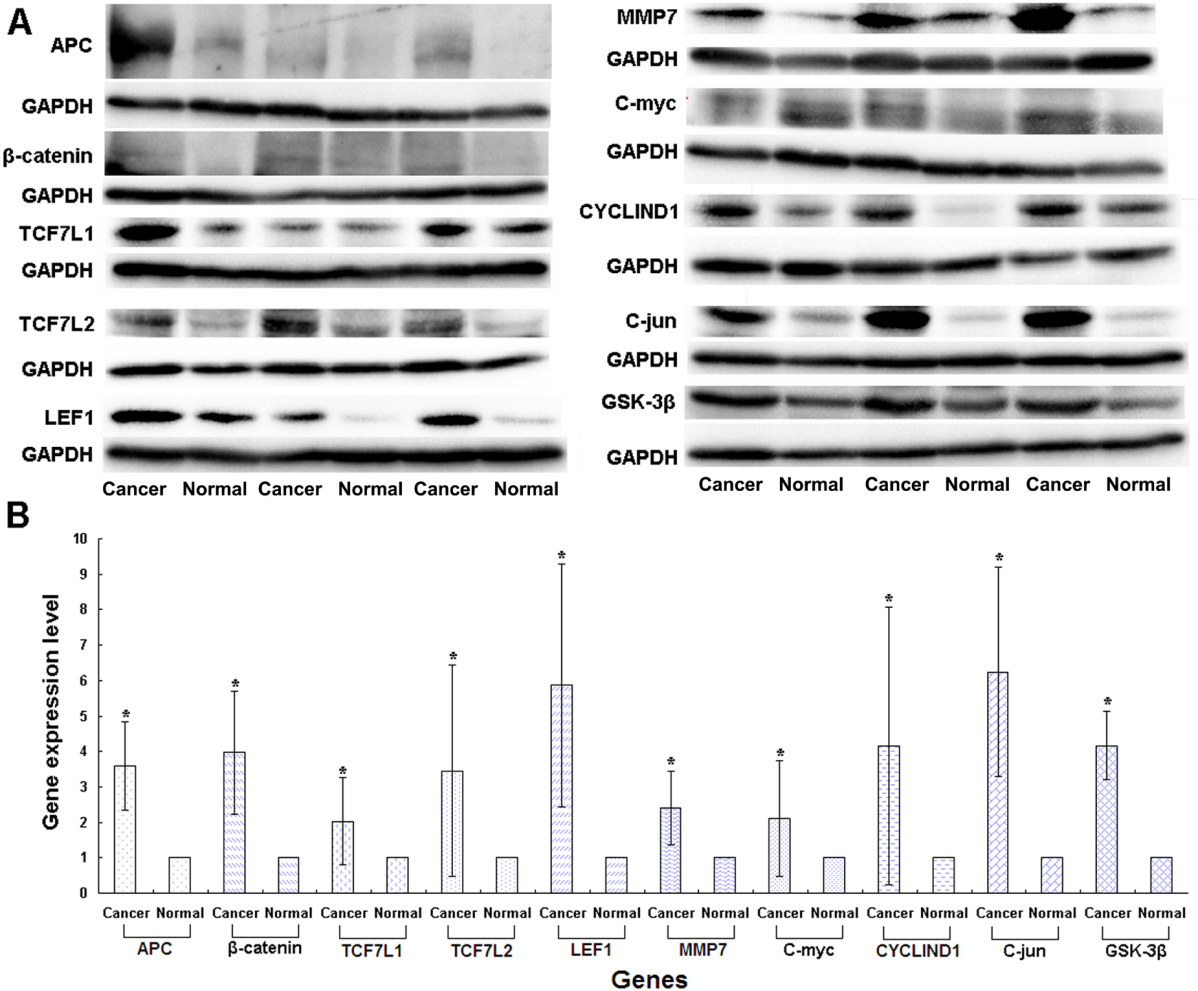
Expression levels of genes in the Wnt/β-catenin signaling pathway in CRC patients detected in the p.1125Val>Ala mutant FAP family patients by Western blotting **(A)** Original experimental results; **(B)** Numerical experimental results with the band values in the original experimental pictures read by the image J software. The protein expression levels were normalized to GADPH.

**Figure 2:**
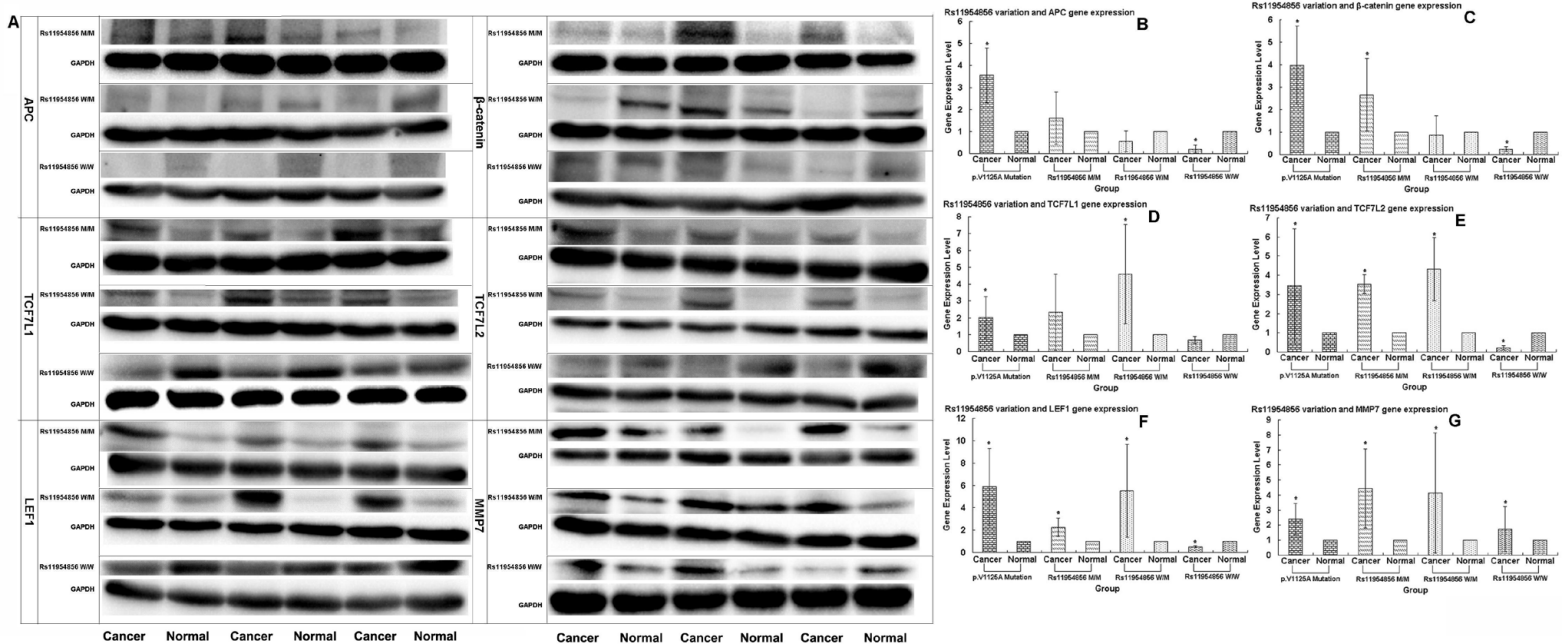
Expression levels of *APC, β-catenin*, *TCF7L1*, *TCF7L2*, *LEF1* and *MMP7* genes detected in patients with rs11954856 wild type or heterozygous or homozygous variations by Western blotting **(A)** Original experimental results; **(B, C, D, E, F, G)** Numerical experimental results with the band values in the original experimental pictures read by the image J software. The protein expression levels were normalized to GADPH.

**Figure 3:**
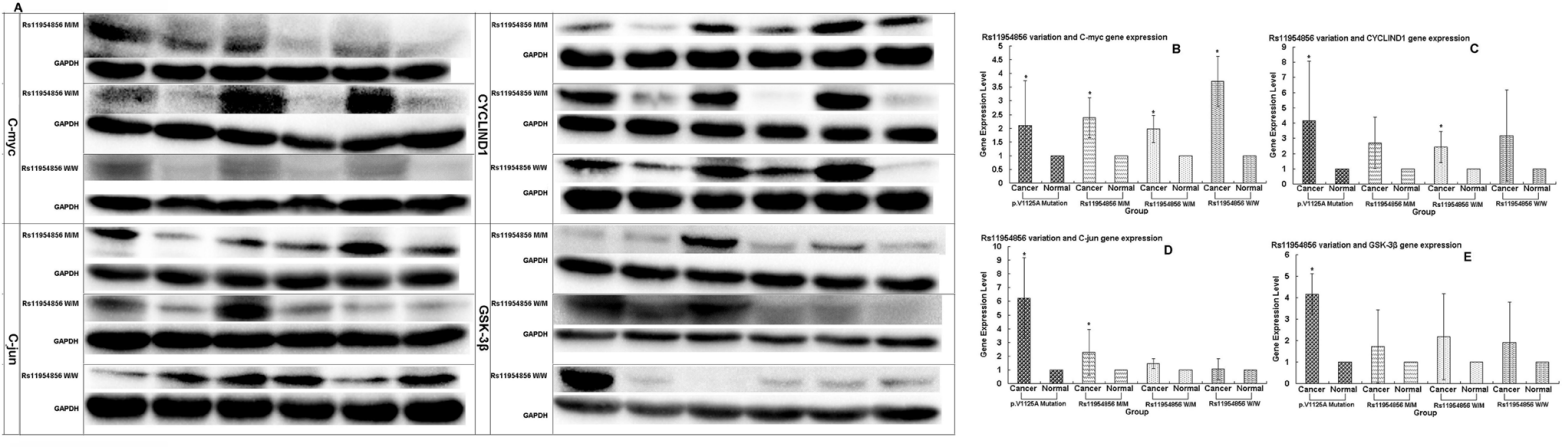
Expression levels of *C-myc, C-jun, CYCLIND1* and *GSK-3β* genes detected in patients with wild type or heterozygous or homozygous variations by Western blotting **(A)** Original experimental results; **(B, C, D, E)** Numerical experimental results with the band value in the original experimental pictures read by the image J software. The protein expression levels were normalized to GADPH.

We used Western blotting analysis to measure the expression levels of *APC* gene and related genes in the Wnt/*β*-catenin signaling pathway, including *β-catenin*, *TCF7L1*, *TCF7L2*, *LEF1*, *MMP7*, *C-myc*, *C-jun*, *CYCLIND1* and *GSK-3β* in both cancer and normal tissues, for the patients who may have the wild or mutation types of the genes, the latter being either heterozygous or homozygous. We used the p.1125Val>Ala mutation in the *APC* gene, which is associated with the FAP syndrome [32], for the positive control. We found that the expression levels of the genes in the Wnt/β-catenin signaling pathway, including *APC*, *β-catenin*, *TCF7L1*, *TCF7L2*, *LEF1*, *MMP7*, *C-myc*, *C-jun*, *CYCLIND1* and *GSK-3β*, were remarkably higher in cancer than non-cancer tissues in the p.1125Val>Ala mutant FAP family members (Figure 1A, 1B). Of significant importance, homozygous variation of the *APC* gene SNP rs11954856 was associated with higher expression levels of the *APC* and*β-catenin* genes in the cancer tissue (Figure 2A, 2B, 2C). Notably, in patients with wild type or heterozygous variation types, the expression levels of the two genes in the normal tissue were higher than those in the cancer tissue (Figure 2A, 2B, 2C). The expression levels of cell cycle proteins MMP7, C-myc, C-jun, CYCLIND1 and β-catenin degradation protein GSK-3β in the Wnt/β-catenin signaling pathway were higher in cancer than in non-cancer tissues in all the wild type and the homozygous and heterozygous variations of the patients (Figure 2A, 2G and Figure 3A, 3B, 3C, 3D, 3E). Conversely, the expression levels of transcription factors TCF7L1, TCF7L2 and LEF1 in the Wnt/β-catenin signaling pathway were higher in cancer tissue in both homozygous and heterozygous variation types of the patients (Figure 2A, 2D, 2E, 2F).

### Comparative analysis of clinical features

We also compared the clinical characteristicsbetween the wild type, heterozygous variant and homozygous variant groups of the CRC patients. We found statistically significant differences between the three groups in stool occult blood and CA199 but not in other clinical characteristics, such as gender composition, age, white blood cell counts, CEA, TNM stage, tumor sites, or pathological types and grades etc. (Table 6). Multiple testing assays on the three groups demonstrated statistically significant differences between the wild type, heterozygous, and homozygous variant groups of the CRC patients in stool occult blood (Table 6). For CA199, however, we found statistically significant differences only between the wild type and heterozygous variant groups (Table 6).

**Table 6:** Comparative analysis of clinical features between wild type, heterozygous variation and homozygous variation groups

Clinical Index	Wild Type	heterozygous variation	homozygous variation	Chi-Square test
**Gender (Male/Female)**	105/74	65/40	13/3	P=0.201
**Blood in stool (Yes/No)**	119/60	56/49	5/11	P=0.005
**BSMT**	WT-HE P=0.028	HE-HO P=0.100	WT-HO P=0.005	——
**Smoking (Yes/No)**	64/115	35/70	6/10	P=0.897
**Drinking (Yes/No)**	59/120	45/60	8/8	P=0.140
**Type (Rectum/Colon)**	97/82	53/52	11/5	P=0.384
**Age**	59.64±12.09	59.24±12.79	55.09±15.35	P=0.512
**White Blood Cells**	6.69±2.35	6.71±2.27	7.83±2.72	P=0.299
**NGP**	61.54±10.94	60.73±10.04	64.96±14.33	P=0.474
**Hemoglobin**	126.81±26.31	127.69±23.53	130.64±16.61	P=0.876
**Platelets**	251.02±86.04	242.45±72.04	244.64±74.08	P=0.760
**ALT**	16.24±10.23	17.19±11.32	14.36±5.95	P=0.652
**AST**	18.77±7.78	18.49±8.07	16.00±4.86	P=0.524
**Prealbumin (SPA)**	180.68±75.07	171.99±64.97	192.30±70.56	P=0.569
**Albumin**	40.51±6.42	44.85±41.60	40.13±4.97	P=0.471
**Creatinine**	75.77±19.03	76.71±17.87	80.57±33.00	P=0.720
**CEA**	17.92±87.83	21.04±71.96	3.58±3.06	P=0.769
**CA199**	36.95±133.43	107.30±279.63	14.05±12.97	P=0.034
**CA199MT**	WT-HE P=0.013	HE-HO P=0.138	WT-HO P=0.707	——
**TNM Stage (I/II/III/IV)**	25/85/58/11	6/56/38/5	0/12/4/0	P=0.115
**Tumor Sites (left/right)**	133/46	73/32	15/1	P=0.117
**Pathological Types (PU/PP)**	97/82	65/40	10/6	P=0.408
**Pathological Grades (H/M/L/MA)**	21/148/4/6	9/88/4/4	4/12/0/0	P=0.522

NGP: neutrophilic granulocyte percentage; BSMT: blood in stool-multiple testing; CA199MT: CA199-multiple testing; PU: pathological ulcerative type; PP: pathological protuberant type; H: pathological high grades; M: pathological moderately grades; L: pathological low grades; MA: mucinous adenocarcinoma.

## DISCUSSION

**Figure 4:**
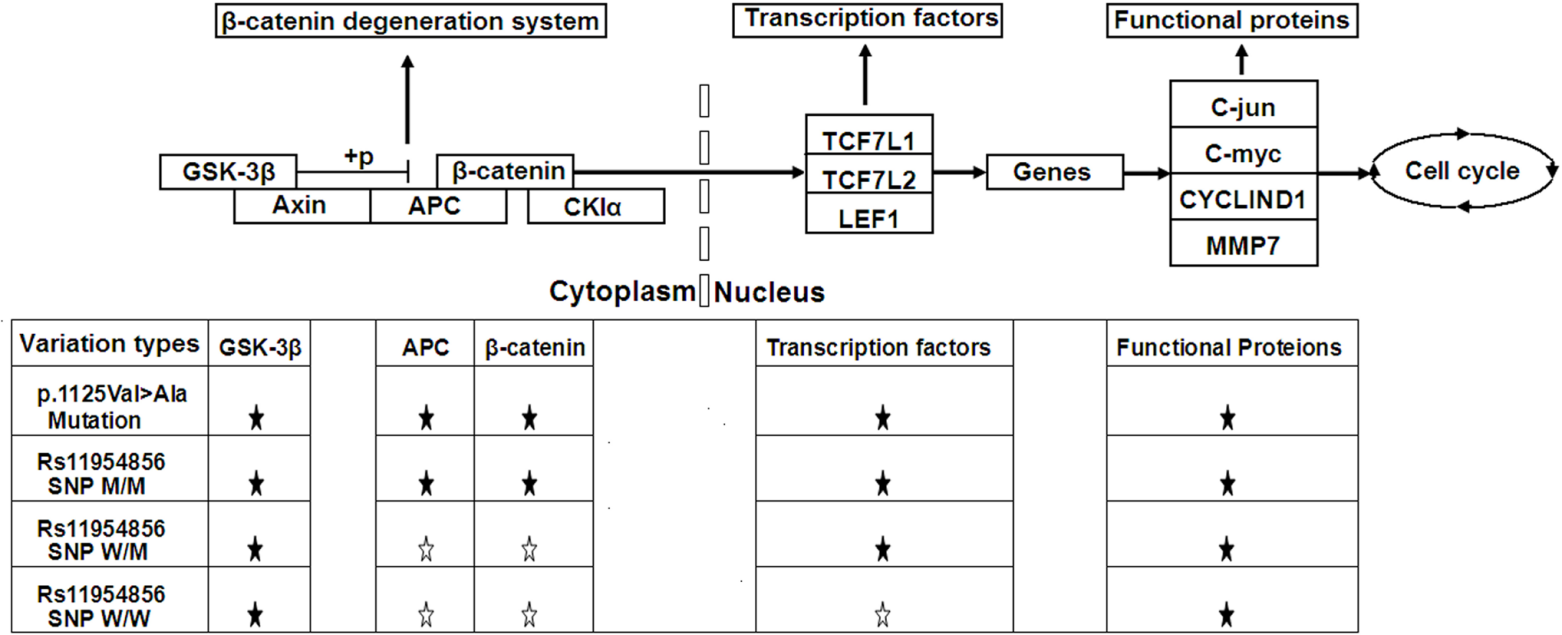
Schematic diagrams of Wnt/β-catenin signaling pathway Shown here are influences of rs11954856 and the p.1125Val>Ala mutation in *APC* gene on the expression levels of genes in the pathway in CRC patients. ★ denotes statistically significant and ☆ denotes no statistical differences.

In this study, we found that the *APC* gene SNP rs11954856 was associated with colorectal cancer and increased the expression levels of genes in the Wnt/β-catenin signaling pathway in the CRC patients (Figure 4). Of remarkable significance, the p.1125Val>Ala mutation in the *APC* gene, previously reported for its association with the FAP syndrome [32], also increased the expression levels of all the genes downstream of *APC* gene in the Wnt/β-catenin signaling pathway in the CRC patients, including *APC*, *β-catenin*, *TCF7L1*, *TCF7L2*, *LEF1*, *MMP7*, *C-myc*, *C-jun*, and *CYCLIND1*, and *GSK-3β* for feedback located upstream of the *APC* gene (Figure 4).

As an important integral part of the Wnt/*β*-catenin signaling pathway, the protein encoded by the *APC* gene plays key roles in tumor suppression by antagonizing the pathway [23]. Loss of APC functions can lead to inappropriate activation of the Wnt/*β*-catenin signaling pathway and thus may facilitate carcinogenesis [35]. Additionally, APC also has important roles in cell functions, such as migration, adhesion, chromosomal segregation, spindle assembly, apoptosis and differentiation [23]. The sequence about 40 kb upstream of the initiator codon is the promoter of the *APC* gene [36] and its hypermethylation is associated with dysfunction of the Wnt/*β*-catenin signaling pathway [37]. Transcripts bearing the untranslated exon of *APC* are spliced to either exon1 or exon2, which can be detected in mouse embryonic stem cells and all mouse tissues, demonstrating the housekeeping feature of these sequences [36]. We found in this study that the SNP rs11954856, located before the exon1 and within the 3,261 site upstream of the initiating methionine of the *APC* gene, was associated with colorectal cancer.

Compared to the p.1125Val>Ala mutation in the *APC* gene, which increases the expression of not only the genes downstream of the *APC* gene in the signaling pathway but also the *GSK-3β* gene upstream of the *APC* gene, rs11954856 increased the expression of only *APC* and *β-catenin* in the CRC cancer patients, with lesser influences on those of the transcription factors *TCF7L1*, *TCF7L2* and *LEF1*. The amino acids from 1265 to 2035 in the APC protein form the domain for bindingβ-catenin, which is important for β-catenin degradation [38, 39]. Since the variations in the binding domain may increase APC binding withβ-catenin, this domain has been believed to have key roles in the pathogenesis of CRC especially FAP [40]. The results of the present work provided new support for the involvement of APC in the pathogenesis of CRC.

The distinct influences between the SNP rs11954856 and mutation p.1125Val>Ala on the expression levels of genes in the Wnt/β-catenin signaling pathway in the CRC cancer patients may be associated with their different locations in the *APC* gene or different features of particular SNPs/mutations or both. Nucleotide changes may inactivate *APC* gene and in the meantime activate or stabilize *β-catenin*, resulting in the permanent activation of the Wnt/β-catenin signaling pathway, and the silenced *β-catenin* may lead to decreased colonosphere formation, which is an important feature in the tumorigenesis [41]. When *TCF/LEF* and *C-myc* are hyperactivated, the formation of colonospheres is increased [42]. Such events in stem cells may result in increased tumorigenesis and cause CRC [43]. Our findings in the present work are consistent with the previous reports [41–43], such as the *APC* gene p.1125Val>Ala mutation increasing the expression levels of all the genes in the Wnt/β-catenin signaling pathway in the CRC cancer patients (Figure 4).

The main features of the gastrointestinal epithelium cells include rapid cell proliferation and differentiation [43], so mutations may accumulate quickly, with some contributing to carcinogenesis. Additionally, other factors, such as advanced age [44], smoking [45], unhealthy lifestyle and polluted environment also play important roles in the pathogenesis of CRC syndrome [6]. The genes in the Wnt/β-catenin signaling pathway are affected by multiple genetic factors. For example, the expression of *C-myc* can be suppressed by hyperactivation of cell checkpoint genes such as *CDKN1A*, *CDKN1B* and *CDKN2B* [46, 47]. Therefore, the increased expression levels of cell cycle functional proteins MMP7, C-myc, C-jun and CYCLIND1 in the Wnt/β-catenin signaling pathway that we observed here in the cancer tissue (Figure 4) may have contributions also from other genes or factors.

In conclusion, we found that the *APC* gene SNP rs11954856 was associated with CRC by increasing expression levels of genes in the Wnt/β-catenin signaling pathway in the CRC cancer patients, compared to the p.1125Val>Ala mutation in *APC*, which increased expression levels of not only all the genes downstream of *APC* in the Wnt/β-catenin signaling pathway in CRC but also *GSK-3β* upstream of *APC*. Recently, some researchers have found that the SNP rs11954856 is associated with the risk of ovarian and gallbladder cancers [48, 49]. All such findings indicate the importance of the SNP rs11954856 in pathogenesis of cancer and may help develop new strategies for the treatment of cancers or new biomarkers for evaluation or diagnosis of colorectal cancer.

## MATERIALS AND METHODS

### Study population

We included 300 sporadic CRC cases and 411 normal controls (Supplementary Table 1) for this study, which were assembled at the Department of Colorectal Surgery and Medical Examination Center of the Second Affiliated Hospital of Harbin Medical University, Harbin, China. We obtained a written informed consent from each participant or guardian, and this work has been reviewed and approved by the Ethics Committee of Harbin Medical University. We also confirmed that all experiments were performed in accordance with relevant guidelines and regulations, consistent with the 1975 Declaration of Helsinki. Medical histories were recorded in detail for all the enrolled participants, and all participants received physical and enteroscopic examinations.

### DNA analysis

We extracted genomic DNA from peripheral blood leukocytes of each participant using standard protocols [50]. The *APC* gene was amplified by polymerase chain reaction (PCR) with the primers (Supplementary Table 2) for analysis of SNPs. PCR products were sequenced using standard protocols [51] for genotype analysis.

### APC SNP genotyping and statistical analysis

The variations of rs11241185, rs11954856, rs2019720, rs2229992, rs2431238 and rs2289484 within the *APC* gene (Supplementary Figure 1A) were determined for 300 sporadic colorectal cancer cases and 411 normal controls. We amplified the *APC* gene and sequenced the PCR products to determine the genotypes (Supplementary Figure 1B).

The statistical analyses were conducted using the SPSS software (version 19.0) and PLINK v1.07 software (http://pngu.mgh.harvard.edu/Bpurcell/plink/) by the methods as previous reported [52, 53]. P values less than 0.05 were considered statistically significant. The Hardy-Weinberg equilibrium test of the CRC and control populations was conducted with the online software OEGE [54].

### Western blotting analysis

Proteins of the tumor and normal tissues near the tumor were extracted using standard protocols, and the contents were determined by the BCA protein assay kit (from BOSTER) and ELISA. The proteins were separated by 8% SDS-PAGE and transferred to PVDF membrane. The membranes were then incubated with the primary antibodies against the proteins, including APC (No.ab58, Abcam, Cambridge, USA), β-catenin (No.ab32572, Abcam, Cambridge, USA), TCF7L1 (No.ab133360, Abcam, Cambridge, USA), TCF7L2 (No.ab76151, Abcam, Cambridge, USA), LEF1 (No.ab137872, Abcam, Cambridge, USA), C-myc (No.sc40, Santa, California, USA), C-jun (No.ab32137, Abcam, cambridge, USA), CYCLIND1(No.ab134175, Abcam, cambridge, USA), MMP7 (No.ab205525, Abcam, cambridge, USA), GSK-3β (No.sc53931, Santa, California USA) and GAPDH (No.ta08, ZSGB-BIO, Beijing China) in 5% non-fat milk in TBST at room temperature for two hours. After washing for three times using TBST, the membranes were incubated with secondary antibodies (No.zdr5306 and 5307, ZSGB-BIO, Beijing China) at room temperature for two hours. Then the membranes were developed using the enhanced chemiluminescence plus reagent and imaged using the Bio-Rad gel imaging system [55]. Finally, the band values were read using the image J software.

## SUPPLEMENTARY MATERIALS FIGURE AND TABLES


