# Supplementary material for: Different effection of p.1125Val>Ala and rs11954856 in APC on Wnt signaling pathway
Source: Oncotarget. 2017 Aug 5;8(41):70854–64. doi: 10.18632/oncotarget.20106 (PMC5642601; doi:10.18632/oncotarget.20106)
Supplement: Supplementary file 1 [file oncotarget-08-70854-s001.pdf]

Different effection of p.1125Val>Ala and rs11954856 in APC on Wnt signaling pathway

SUPPLEMENTARY MATERIALS

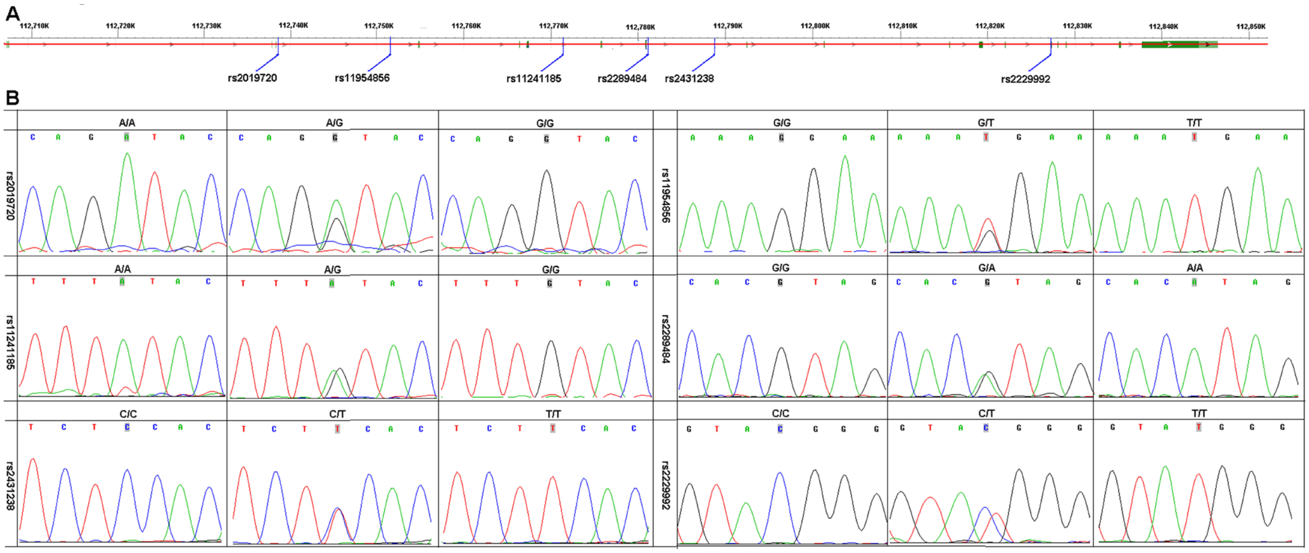

Supplementary Figure 1: Schematic diagrams and DNA sequence chromatogram of SNPs in *APC* gene. **(A)** locations of rs11241185, rs11954856, rs2019720, rs2229992, rs2431238 and rs2289484 within the *APC* gene; **(B)** DNA sequence chromatogram of the three polymorphisms identified in the *APC* gene in all the population used for disease-association analyses.

Supplementary Table 1: Clinical characteristics of study population

Parameter	*CRC*	*Control*	*F*	*t*	*P*	*95%CI*	
						Up	*Low*
Sample (n)	300	411	-	-	-	-	-
Male/Female (n)	183/117	256/155	-	-	0.727	-	-
Age (years)	58.59±12.59	59.39±4.07	60.443	-0.613	0.541	-3.37998	1.77645

Data are shown as mean±SD; between the two groups, there were no statistical differences of the age and gender composition.

Supplementary Table 2: PCR primers used for SNP analysis in the *APC* gene sequence

SNPs	*Forward primer*	*Reverse primer*	*Size (bp)*	*Tm (°C)*
rs11241185	AGATGATGCTATTTGGAC	GTACCTTGATAATGAAGAA	408	44.8
rs11954856	AAGTTAGCTTAGGGAGAA	ATGTGGAAACACTGGATA	437	44.0
rs2019720	CAGAGCGTGAGTGGTGGT	GGCCTAACAGAGGGAGAA	193	51.1
rs2229992	TTTTCCTAGTATTTAAGTTACC	CTTGTTGGCTACATCTCC	226	46.6
rs2431238	CTCAGAACCTGGCACATA	CAGGAAGACTGCTGGATA	246	44.2
rs2289484	TCAGAGTTGCGATGGAAG	CAAGGCAGAACAGAACAG	285	74.1
